# Thoughts on the Pathology and Treatment of Cynanche Trachealis, or Croup

**Published:** 1821-05

**Authors:** N. Chapman

**Affiliations:** Professor of the Institutes and Practice of Physic and Clinical Practice, in the University of Pennsylvania.


*Thoughts on the Pathology and Treatment of Cynanche Trachealis, or Croup.* By N. Chapman, m.d. Professor of the Institutes and Practice of Physic and Clinical Practice, in the University of Pennsylvania.

TO this affection various other names have been applied, by the different writers who have treated of it. It is called suffocatio stridula, angina polyposa, asthma infantum, cynanche stridula, angina epidemica, morbus strangulatorius; and, in popular language, croup, or hives, the heaving of the lights or lungs, the choak or stuffing, &c. The best nosological title is *tracheitis:* it clearly designates the more ordinary nature of the complaint, and at the same time gives uniformity to our medical nomenclature.

Croup has commonly been considered as a disease of modern date, and the credit of having originally noticed and described it is accorded to Professor Home, of Edinburgh, whose publication appeared about the middle of last century.* Turning over, however, one of the earliest volumes of the Transactions of the Royal Society of London, I find a very distinct account of the disease, illustrated by dissections. The writer, who was an obscure practitioner, describes it as an entirely new complaint, which had suddenly appeared among the children of Cornwall, committing very considerable ravages. It is also said to be particularly noticed by Martin Ghisi, an Italian writer, so early as 1749.†

Croup is, for the most part, confined to the early period of life, embracing the space between the first and fifth year, and affects chiefly children florid and robust. But I have known it to attack infants within the month, and also adult subjects. The illustrious Washington is said to have died of this disease. Two ladies of this city, who are now nearly in the meridian of life, I have attended in repeated attacks of croup: so strongly, indeed, are they predisposed to it, that they scarcely ever escape when exposed to the causes. The same liability has been transmitted to all their children, who are now numerous.

By some writers, however, it is asserted, that croup never occurs after the age of puberty. That it is a rare event, cannot be denied; nor, perhaps, is the fact without explanation. The parts constituting the seat of the disease undergo at this period a change, as is evinced by the new tone of voice acquired; which change enables them to resist those causes that, in the previous state of debility and relaxation of the larynx, more especially were invited to such morbid aggressions. Cases of this kind, however, are still to be considered as rare and anomalous deviations from the ordinary course and character of the disease.

Notwithstanding what has been so confidently alleged to the contrary, there is not the slightest reason to believe that croup is ever propagated by contagion. It would seem chiefly to arise from the influence of a moist and cold or austere atmosphere; and hence prevails more generally in the spring than at any other season, and near to the sea or other large collections of water, rather than in inland positions.

By some writers it is affirmed occasionally to occur as an

* 1765.

† There is, indeed, some reason to suspect that several of the much older authorities meant this disease, in the descriptions which they contain of a very fatal species of angina, *without swelling of the throat.*

epidemic, and perhaps this may be true. It is certain that the complaint is endemial to particular places, and within very narrow limits. Many situations on the sea-board are so particularly exposed to it, as almost to preclude the raising of children.

Croup has been divided into spasmodic and inflammatory, and not a little discussion has taken place on this subject. It would seem to me that, in all cases where it suddenly attacks, it must partake of the nature of spasm. Time is required to induce inflammation, which consists in an altered action of the vessels of a part, affected by comparatively a slow process. No cause, however, more rapidly promotes it than the disturbance occasioned by spasmodic constriction.

The early symptoms correspond with this view of the pathology of croup, and dissections fully confirm it, showing, where death promptly takes place, none of the phenomena of inflammation. But, under other circumstances, where the disease slowly approaches, or is the effect of inflammation of other parts, extending to the trachea, as sometimes happens in measles, scarlet fever, and most of the anginose affections, then it is of a contrary character; and *post mortem* inspections have revealed exactly such appearances as might have been anticipated.

Even, however, admitting the distinction contended for, I am not aware that it leads to any practical difference. Whether spasmodic or inflammatory, the directly depleting measures will be found equally effectual in the treatment. No remedy is so prompt in the reduction of spasm of high action as venesection, and none so unavailing or inappropriate as the antispasmodic substances. On this point I wish to speak emphatically, since some of the European as well as our own writers of high authority, entertaining other notions, have laboured to establish an opposite practice, consisting in the use of musk, assafœtida, and opium; than which nothing can be more false or prejudicial.

Croup variously makes its attacks. It commonly comes on at night, and sometimes without any premonition or exposure to its ordinary causes. The child wakes up with the hoarse, dry, stridulous cough, peculiar to the disease; which has been aptly compared to the sharp sound of the barking of a dog, and in other instances to the crowing of the cock. Concomitant with this, there is a distressing difficulty of respiration, menacing, in some instances, suffocation; with a flushed face, a quick irritated pulse, an unusual degree of restlessness and anxiety, wish a sort of indescribable wretchedness. The child will not remain long in one position, nor can its complaints be in any way appeased. It whines, and cries, and frets, and seems to be excessively uneasy, without suffering any very positive pain.

Cases of this nature are probably dependent on spasm, and terminate fatally in a very short time, where relief is not afforded. But, on many occasions, the disease advances gradually, with the ordinary catarrhal symptoms,—such as heaviness, suffusion of countenance, defluxions from the eyes and nose, a harder and more shrill cough than usual; and with various degrees of fever, which, with the cough, is always exacerbated at night, and especially after the child has slept. Completely formed, there is no material difference between the two species of croup; and henceforward their progress is nearly, or perhaps exactly, similar.

My mode of managing this disease is exceedingly simple, and has hitherto proved so successful, that I always approach it, in the early stages, with a greater certainty of curing it than any of the other complaints of infancy or childhood.

Called in the commencement of the attack, I endeavour at once to puke the child very freely; and for this purpose prefer the tartarized antimony, given at short intervals, as being one of the most certain and powerful of the emetics. At the same time I direct the child to be put into a warm bath for ten or fifteen minutes. This is a useful remedy: it rarely fails to promote the operation of the emetic, and will, indeed, alone sometimes cure the disease. The emetic, however, not operating,—or, if after its operation, the desired effect be not realized,—I then bleed copiously, and repeat it and the bath. An attack must be extremely obstinate if it do not now yield. Nevertheless, it will occasionally continue with little or no abatement; and, under these circumstances, I resort to topical depletion, by leeches or by cups. The cups should be applied to the sides or back of the neck; as, when placed anteriorly, they will, by pressure and suction, greatly impede respiration, and sometimes endanger suffocation. Twice I have seen the distress from this mistake so violent, that I believe death would have taken place had not the cups been removed. As means of local bleeding, leeches are very much to be preferred in such cases. Next I put a sinapism or blister over the throat; or, in some instances, these may be made to precede the former applications.

The foregoing remedies failing, or where the symptoms become so alarmingly violent as to demand immediate relief, I bleed *ad deliquium animi.* When pushed to this extent, I may almost say that venesection is invariably successful; as yet, I have never known one instance in which it failed. The moment that syncope takes place, the coarseness, cough, impeded respiration, and fever, disappear.

This valuable suggestion I derived from Dr. Dick, of Alexandria, one of the most original, bold, and successful practitioners of our country. It has been claimed, I understand, elsewhere, with what justice I pretend not to determine. That, however, the practice was adopted at least thirty years ago, by this distinguished physician, is unquestionable.

To prefer small and repeated bleedings at this period of the disease, as is advised by one of the most authoritative of our own writers, is a pernicious abuse of an important remedy. It may be laid down as a rule to which there are few exceptions, that, in acute diseases, where venesection is at all demanded, it should in the commencement be so copious as to produce decisive effects. The rationale of the measure seems not to be well understood. Detractions of blood, in a small or large quantity, operate, as remedial processes, very differently: the former *abates* action only; white the latter *alters* it, or so far reduces it as to enable the natural energies of the system to subvert or overcome it, and to re-establish health. Of this principle we have illustrations in pleurisy, in fevers, and many other affections, where a single profuse bleeding, timely recurred to, arrests the progress of the case.

Conceding that the loss of blood is necessary to a cure, it will be proper, under the circumstances stated, to pursue this course, even where we have grounds to apprehend debility. As small bleedings require to be often repeated, the aggregate of blood lost becomes ultimately greater, and more exhausting in its effects. Besides which, as there is less structural or functional derangement, the convalescence is more rapid and complete. Whether, therefore, with a view to a prompt cure, or to economize the resources of the constitution, or as a security against relapses or imperfect recoveries, this practice claims a preference.

The disease being broken, which is shown by the removal of the preceding symptoms, and even still more by the restoration of the natural susceptibility of the system to the action of medicine, I administer calomel, not in small and repeated doses, as is more generally advised, but in the largest possible dose, in order that it may speedily and most actively purge. In this particular stage of the disease, a thorough opening of the bowels carries off the lingering symptoms, obviates a relapse, and confirms the convalescence. But, should cough or hoarseness, with tightness of the chest and deficient expectoration, remain, I employ the polygala senega as an expectorant. It is in extinguishing the remains of croup, that it displays, I think, not the least of its valuable properties. Doubtless, however, it may be used at an earlier period of the disease with advantage as an emetic; though still I prefer the tartarized antimony.

The practice, as here detailed, is applicable chiefly to croup in its forming and early stages. At this period the disease is restricted pretty much to the upper portion of the trachea, and consists either in a spasmodic constriction of the glottis or inflammation of the membranous lining of the larynx: but, permitted to continue for ten or fifteen hours, and sometimes even in a shorter interval, it extends itself to the bronchiæ and into the substance of the lungs, producing, sooner or later, vast collections of mucus and phlegm, or exudations of coagulable lymph, or an engorged state of the pulmonary organs with blood.

The symptoms at this critical conjuncture are materially different. Now we have all the manifestations of an interrupted and defective circulation. The lungs, loaded and oppressed, very imperfectly execute their functions. The complexion is mottled, and the cheeks have a circumscribed flush, with some mixture of lividness. The eyes are prominent and inflamed. The pupil is often widely dilated; attended by an expression of countenance wild, haggard, and ghastly. The respiration is exceedingly laborious, with a full and disturbed pulse; or the child, sinking under the disease, has its breathing rather more tranquil, with a weak and irregular circulation.

The symptoms in these different states of the lungs are so analogous, that it is not easy to establish, in all instances, satisfactorily, a diagnosis: but, though difficult, it is a point of some consequence to be determined, as the treatment in every respect is not precisely the same. To arrive at a just conclusion, we must take into view all the circumstances appertaining to the case in its several stages, as well as the existing appearances.

Of the nature of bronchitis, and especially of that form of it which resembles catarrhus suffocativus,—or, in other words, when it proceeds from collections of phlegm, or mucus, or lymph, in the bronchiæ or pulmonary cells,—I have mostly found that the case has had its origin in catarrh, and which has run a course more than ordinarily protracted. There is also at the time greater or less discharge from the lungs, or at least evidence of heavy accumulations of matter, with an inability to throw it up; and to which may be added, that the pulse is languid, and the surface cold and clammy. But, occasioned by sanguineous congestion, however oppressive the dyspnœa may be, there is little or no cough or pituitous discharge; and, what is very distinctive, an entire absence of the wheezing, so general a symptom in the first case. The respiration, however, is singularly hurried, panting, and laborious. The pulse, too, is full, though irregular and disturbed, and very readily compressible. Cases of this sort, moreover, are apt chiefly to occur in florid and plethoric children; or, as I have seen, in directly the reverse, the weak and valetudinary; and generally this condition is disclosed at an earlier period in the disease.

The indication now, in each shape of croup, is to relieve the lungs of oppression, and to re-establish a free and equable circulation. To effect these purposes, the child should be placed in a warm bath, and, while there, copiously vomited by an active and stimulating emetic. The sulphate of zinc has been recommended, and is useful, though the tartarized antimony, with calomel and ipecacuanha, or the juice of garlic or onion, is preferable.*

But, in the second case, having pursued the same measures, we are also very cautiously to draw blood; taking away a little at once, suppress the flow, and watch the effect on the system. Being beneficial, we may renew the bleeding from time to time, till our views in this respect are attained.

The necessity of such extreme circumspection in the use of the remedy in this case, is readily explained. Engorgement of the great viscera, and especially the lungs, takes out of the general circulation such a large portion of blood, and confines it so closely in the part, that any considerable loss by venesection is very sensibly felt, creating, in some instances, prompt and irreparable exhaustion.

Where the lancet is altogether forbidden, cups or leeches may be substituted, and will be most serviceable on the back.

In each species of the disease, the vesicating applications are highly important remedies. The blister should be put over the breast; or, if the case be so urgent as not to admit of delay, some means of more prompt vesication may be resorted to,—as cloths wrung out of hot water, or, what perhaps is better, pledgets of lint dipped in a decoction of cantharides, made with the spirit of turpentine.

The subsequent treatment consists principally of the pretty constant use of expectorants; and for this purpose the antimonial wine, the oxymel or vinegar of squills, the decoction of Seneka-snake root, either alone or in combination with the carbonate of ammonia, will answer exceedingly well. The hive syrup is here a very useful preparation.†

---

* These latter are very certain and active emetics, and will frequently succeed in exciting vomiting when the officinal articles have failed.

† This is a prescription of Professor Coxe, which I have reason to believe has justly acquired great popularity in the treatment of croup. It is prepared agreeably to the following formula; and the dose is about a tea-spoonful for a child of one or two years old.

*Syrupus Scillæ Compositus.*—℞. Seneka snake-root, bruised; Squills, dried and bruised, ā ℔ss.; Water, ℔8. Boil together over a slow fire, until the water is half consumed; strain off the liquor, and add, Strained honey, ℔ 4. Boil them together to six pounds, or to the consistence of a syrup; add to every pound of this syrup, sixteen grains of tartar emetic,—that is, one grain to the ounce.

See *American Dispensatory*, 4th edit. p. 343.

Much may also be expected, in some instances, from the liberal exhibition of calomel. At all times an exceedingly active expectorant,—by which I mean whatever enables the bronchial structure to disengage and expel an oppressive load of matter, —it seems, under these circumstances, occasionally to operate with really a specific efficacy. There are some, indeed, of the respectable practitioners, both of this country and of Europe, who trust almost exclusively to it.

Calomel was originally employed in croup by the late Dr. Kuhn, of this city, who prescribed it so early as the year 1770. The Scotch physicians are devoted to the remedy, and consider it almost infallible; or such rather seems to be the opinion of some of the most distinguished of their writers. By one of them it is said, "that, in every case where it was employed previous to the occurrence of the lividness of the lips and other mortal symptoms, it has completely succeeded, both in curing the disease and in preventing any shock to the child's constitution." His manner of exhibiting calomel would appear daring, even to rashness, were we not acquainted with the insensibility of the system in this disease to remedial impressions of every description. To a child of two years old, he has given upwards of one hundred grains in twenty-four hours.

With Dr. Hamilton, to whom I have alluded, the professor of midwifery at Edinburgh, I am acquainted, and, from his high standing and character, I entertain not the slightest doubt, with some allowance for an undue enthusiasm of expression, of the veracity of these representations. Nevertheless, I will not take upon myself to support or recommend his practice. The mode which I have suggested of managing this disease, (at least as it appears in this country,) I must think decidedly more effectual, and certainly less hazardous, as well as repugnant, to popular prejudices.

In the preceding history, I have delivered, very concisely, some account of the pathology and treatment of croup. It results from what has been said, that I consider it at first as a spasmodic or inflammatory affection of the larynx; and in its subsequent stages as one or the other of the forms of peripneumonia notha,—either a congestion of the lungs with mucus or lymph, or with blood. The former I believe to be by far the most common occurrence, or usual shape, of the disease.

The practice appropriate to the several circumstances of croup, I have also endeavoured to point out with some degree of precision.

It will be perceived that, in relation to the latter stages of the disease, while I maintain that the lungs are affected differently in some cases, the only distinction in the treatment suggested, is the limitation of bleeding to the apoplectic condition of these organs. Though I hold the other state to be essentially bronchitis, and hence originally of an inflammatory character, still, from the early depletory measures generally pursued, such no longer exists. We have, on the contrary, at this time, as its product, effusions or exudations, obstructing respiration. Yet, wherever there is reason to suppose a remnant of inflammation, topical bleeding, at all events, may be, and ought to be, practised.

What, on the whole, I wish especially to call attention to, is the view which has been presented of the nature of croup at an advanced period. It is interesting, not as mere theory, but as leading to the practical improvement on which I have dwelt. Though not generally entertained or adopted, it is most fully established, as well by the phenomena of the disease already noticed, as by dissections. To this point we have, to a certain extent, the testimony of Cheyne, who has written with ability on the disease, and the still higher authority of Baillie,—not to mention other names of less distinction,—all which has been confirmed by dissections conducted in this country.*

Not a little is said of the existence of a membrane in the larynx, and to which so much is ascribed in occasioning death, that an operation has been proposed, and even practised, for its removal. That it does occasionally exist, cannot be denied, though I suspect rarely, as I never met with it in my repeated examinations for this purpose.

The appearances I have observed in dissections relating to the larynx, were slight marks of inflammation, with more or less of mucus, such as is formed by all the secreting surfaces. Why I have not seen the membranous production, is perhaps susceptible of explanation. To throw out coagulable lymph, of which it is composed, requires the vessels to be highly excited; a state which, by the copious depletion adopted in the cases that came under my notice, was probably prevented.

---

* Neither of these writers, however, have noticed the apoplectic state of the lungs. The venerable Dr. Bard, of New-York, who was among the very first to adopt a correct pathology of croup, says, in his Essay on the subject, published in the year 1771, that he has found the pulmonary organs so dense and solid, from sanguineous congestion, that they exhibited the appearance of the structure of the liver.

Baillie, in his Morbid Anatomy, tells us, "that, when the inner membrane of the trachea is inflamed, it is sometimes lined with a layer of a yellowish pulpy matter. This does not adhere very firmly to the inner membrane, but may be easily separated. It extends from the upper part of the cavity of the larynx into the small branches of the trachea, which are distributed through the substance of the lungs. There is, at the same time, a good deal of mucus in the trachea and its branches, together with a mixture of pus. This is the appearance of the inside of the trachea, in patients who have died from the croup." Cheyne's account of the *post mortem* appearances in the disease so closely resembles the preceding, that I consider it unnecessary to recite it.—*Vide his Essay on Diseases of Children*, Edinburgh, 1801.

Even, however, were we assured of its existence, I do not know that, in ordinary cases, the operation would do more than protract life. The disease at this time has reached the lungs; and hence no relief in this way could be expected. Yet it does sometimes happen, though seldom, that it is restricted to the larynx, and that respiration may be so obstructed by the membrane alluded to, or from an accumulation of mucus, as to threaten the immediate extinction of life. By the removal of the mechanical impediment, an operation might be useful, and has actually proved so in two cases recorded in the foreign Journals, in which relief was instantly afforded, and ultimately recoveries took place. I have now in my possession a drawing executed by my friend the late Professor Dorsey, representing the membrane in a position completely to intercept the passage of air to the lungs; and which, had it been displaced by an operation, as he strenuously proposed, the child would probably have been preserved.

In the estimate of this resource of our art, we ought, moreover, not to overlook the fact of the striking effect, in many cases, from the expulsion of the membrane by vomiting or coughing, and sometimes in a state of things, too, the most critical and alarming. Yet it seems that the operation, on the whole, is deemed a very desperate and precarious expedient, to be held in reserve only for the extremest emergencies, and where common measures have altogether failed.

Two causes have conspired to render croup, which is not necessarily a fatal disease, so much so, that it is placed by some writers even among the *opprobria medicorum*, and by most practitioners is considered a highly obstinate and dangerous affection. The first is an erroneous notion regarding its pathology; and the second, the careless and feeble mode in which it is commonly managed.

An impression almost universally prevails, that children, owing to an extreme delicacy and frailty of constitution, cannot bear any very vigorous impression from remedies. As a natural consequence of such an opinion, the general practice in their complaints is extremely inert; exactly, indeed, of that kind which has been facetiously described as observing a strict neutrality between the patient and the disease, neither declaring for the one nor the other.

By no narrow or partial observation, I am thoroughly persuaded that the very contrary of this opinion is true. Children, I have remarked, display an uncommon tenacity of life and strength of constitution. They often survive under circumstances which destroy adults. They have been found living at the breasts of their mothers who had perished by exposure to cold, as is recorded by travellers and other writers. They confessedly resist contagion better than grown people; and, when attacked, more certainly recover, not only from diseases of this description, but from all others, when properly treated.

Nor is this all. They sustain better the operation of the most active remedies, as vomiting, purging, sweating, blistering, and, I add without hesitation, bleeding.

During the growth of the body, the fluids, in relation to the solids, are larger in proportion, as is distinctly proved. This fulness of their vessels and greater excitability of system, render children peculiarly liable to inflammatory attacks. Nearly all their complaints partake of this character.

It follows, therefore, that they require oftener to be bled; and my own experience convinces me that venesection may be resorted to in their cases with more safety, and decidedly greater advantage. No one who is conversant with their diseases, and has practised venesection much in them, can withhold his assent from the accuracy of this statement.

Endowed with superior vital energies, children have, moreover, very extraordinary recuperative powers. They notoriously recover more speedily from wounds and injuries, and surgical operations; and recruit with greater rapidity, after being reduced either by disease or by remedies of any description.

It is on this account that, while there is any indication of life, however discouraging the appearances may be, we ought never to view the case of a child in an acute disease as altogether desperate; but, still retaining some hope, to continue to minister to the restorative principle of the constitution: and, were this course generally pursued, I am persuaded that we should not unfrequently be rewarded by such cures as reflect lustre on the art, and give to our skill a glorious triumph.

To do this, however, in the disease before us, the practice must be prompt and energetic, and our attendance unremitted till relief is afforded. It is a rule with me never to leave a child, in croup, till the alarming symptoms are over. This great degree of vigilance and attention are necessary, from the rapid career of the disease, and not less from the extreme and peculiar uncertainty of the operation of our remedies in it.

As a most formidable enemy in all its presentations, it should be attacked early, vigorously, and on the very outworks. Delay never fails to invigorate its force; and, when permitted to get possession of the citadel, or, in other words, a firm hold of the system, we shall find it always difficult, and often utterly impracticable, to dislodge it.

